# Synergies of Single Molecule Fluorescence and NMR for the Study of Intrinsically Disordered Proteins

**DOI:** 10.3390/biom12010027

**Published:** 2021-12-24

**Authors:** Samuel Naudi-Fabra, Martin Blackledge, Sigrid Milles

**Affiliations:** Univ. Grenoble Alpes, CNRS, CEA, IBS, F-38000 Grenoble, France; samuel.naudi-fabra@ibs.fr (S.N.-F.); martin.blackledge@ibs.fr (M.B.)

**Keywords:** nuclear magnetic resonance (NMR), single molecule fluorescence, Förster resonance energy transfer (FRET), intrinsically disordered proteins (IDPs)

## Abstract

Single molecule fluorescence and nuclear magnetic resonance spectroscopy (NMR) are two very powerful techniques for the analysis of intrinsically disordered proteins (IDPs). Both techniques have individually made major contributions to deciphering the complex properties of IDPs and their interactions, and it has become evident that they can provide very complementary views on the distance-dynamics relationships of IDP systems. We now review the first approaches using both NMR and single molecule fluorescence to decipher the molecular properties of IDPs and their interactions. We shed light on how these two techniques were employed synergistically for multidomain proteins harboring intrinsically disordered linkers, for veritable IDPs, but also for liquid–liquid phase separated systems. Additionally, we provide insights into the first approaches to use single molecule Förster resonance energy transfer (FRET) and NMR for the description of multiconformational models of IDPs.

## 1. Introduction

Intrinsically disordered proteins (IDPs), that is, proteins without stable, three-dimensional structures, have gained increasing interest in the recent past as their importance within many biological processes has become evident. Indeed, more than 40% of the eukaryotic proteome contains large intrinsically disordered regions (IDRs) [1], and ignoring these flexible parts of the proteins may lead to severe misjudgments of protein function, especially regarding regulatory functions, where IDRs are most prominent [2]. They often work as hub proteins and have more than one interaction partner. To fulfill these tasks, IDPs have developed interaction mechanisms that are very distinct from the classical binding mechanisms employed by folded proteins. Rather than presenting a folded surface with specific physico-chemical properties to their interaction partners, IDPs often only fold or partially fold upon interaction. Such mechanisms are called ‘folding-upon-binding’ or, if the conformation that is bound by the interaction partner is sampled by the free IDP in solution, ’conformational selection’ [3]. However, not all IDPs fold when interacting; many present small linear motifs that interact with their partner through only a few residues [4], and many of these linear motifs (of the same or different kind) can be embedded in the same IDR [5]. Indeed, likely owing to this vast plethora of interaction modes, affinities between IDPs and their (often folded) partners can span several orders of magnitude, reaching from the low nanomolar to even the millimolar regime [6].

A lot of theoretical work has been devoted to characterizing the primary structure of IDPs, which has led to the discovery that polar rather than hydrophobic residues are enriched in IDP sequences, and which now also makes it possible to predict the presence of IDRs with relatively high accuracy [7]. Nowadays, simulations (coarse grained and molecular dynamics simulations) play a large role in attempts to describe the conformational landscape of IDPs [8,9], but a molecular description of IDPs purely from simulations remains difficult. Indeed, comparisons of obtained models with experimental data are important, but the dynamic and flexible nature of IDPs renders them inaccessible to many classical structural biology techniques, such as X-ray crystallography or electron microscopy. In contrast to folded proteins, the structural features of IDPs need to be described by conformational ensembles of rapidly interconverting conformers, rather than single structures. Solution state techniques, such as fluorescence spectroscopy [10], nuclear magnetic resonance (NMR) [11,12], and small angle scattering (SAS) [13], can handle the existence of multiple different conformations in solution as well as the corresponding protein dynamics. Although dedicated tools had to be developed to analyze IDPs, transiently populated folded states, the overall size of the protein, and the time scales of motion have become important parameters for their characterization [14,15,16,17,18,19,20,21,22,23,24,25].

NMR chemical shifts and residual dipolar couplings (RDCs), for example, have contributed to the discovery and analysis of local structural propensities of IDPs, and have been used to construct atomic multiconformational models that describe their conformational landscape [16,26,27,28]. These models have been complemented with SAS, providing information about the overall dimensions of the structural ensemble, and with paramagnetic relaxation enhancements (PREs) to describe longer range contacts (~2.5 nm) [26,29]. Apart from addressing conformational motions of IDPs implicitly through their description with multiple, rather than single, conformations, various NMR parameters can also probe these dynamics explicitly. Spin relaxation, for example, probes fast protein dynamics in the nanosecond time scale, thus investigating the chain dynamics of the unfolded or transiently folded polypeptide chain [19,25]. Relaxation dispersion and chemical exchange saturation transfer (CEST) make it possible to analyze motions on the microsecond to 100s of millisecond time scale [14,30], which can become important upon interaction [23,24,31].

**Figure 1:**
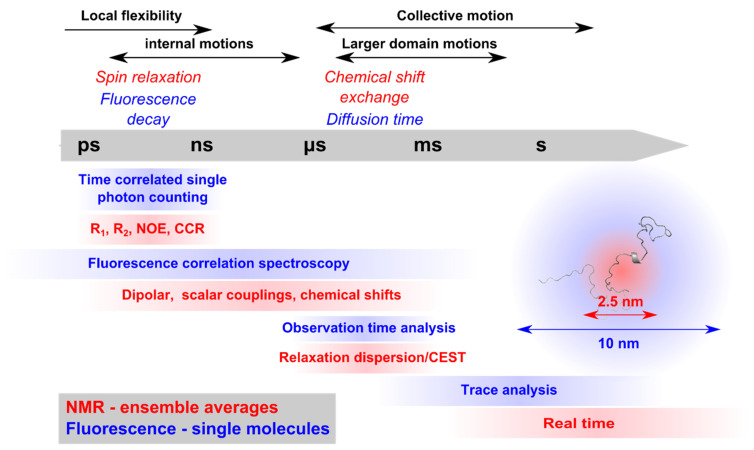
Complementarity of NMR and fluorescence parameters for studying protein conformation and dynamics. Different physical properties determine the time ranges on which NMR and fluorescence spectroscopies are sensitive. Experiments have been designed to probe these ranges and retrieve complementary information on protein dynamics specific to each of the various time scales. Using PREs, NMR distances can reach up to 2.5 nm, while smFRET is sensitive up to about 10 nm. NOE: Nuclear Overhauser effect; CCR: Cross-correlated relaxation; CEST: chemical exchange saturation transfer.

Single molecule fluorescence techniques, in particular single molecule Förster Resonance Energy Transfer (smFRET), provide high resolution information on distances of up to ~10 nm, thereby constituting a powerful partner for the structural analysis of complex and large protein systems containing IDRs (Figure 1). Site-specific labelling of the protein of interest with two fluorophores between which the distance (and dynamics) can be measured makes it possible to study conformationally heterogeneous protein populations when performed on the single molecule level, meaning molecule by molecule [10,32]. Fast dynamics can be inferred with the help of time correlated single photon counting (TCSPC) technologies using confocal detection geometries, and time-resolved fluorescence polarization, as well as fluorescence lifetimes, can supplement intensity-based smFRET, also making it possible to investigate convolved protein distance-dynamics relationships [32]. Using these multiparameter approaches, the dynamics within one state (nanoseconds) and between states (microseconds to milliseconds) can be analyzed in the same experiment. Additionally, the calculation of fluorescence correlation curves from single molecule traces (fluorescence correlation spectroscopy, FCS), for example, has made it possible to investigate time scales ranging from picoseconds to several milliseconds in a quantitative way [18,33], thereby also addressing time scales in the few microseconds/hundreds nanoseconds regime, which are not directly accessible by NMR [34,35].

All these features make single molecule fluorescence and NMR spectroscopy an extremely powerful pair of techniques with which to analyze the conformational dynamics of IDPs. This realization has led to the first studies combining single molecule fluorescence (in particular smFRET) and NMR for the analysis of IDPs. In this review, we provide an overview on approaches that have been performed on folded proteins containing intrinsically disordered linkers, large intrinsically disordered proteins involved in various biological mechanisms as well as those engaged in liquid–liquid phase separation. We will finally shed light onto the first attempts to use information from NMR and smFRET to describe the conformational landscape of IDPs in a quantitative manner.

## 2. Folded Domains Connected by Intrinsically Disordered Linkers

Proteins that are composed of several folded domains often connect their folded domains by intrinsically disordered linkers. In some cases, this does not prevent from structural analysis using, for example, X-ray crystallography. In other cases, however, fast dynamics attributed by the disordered linker can hinder attempts at crystallization, but at the same time is needed for proper functioning of the protein and positioning of the different domains with respect to each other. The benefit of NMR and single molecule FRET to decipher the conformations of multidomain proteins has been demonstrated for a few examples.

One of them is the trans-activation response RNA binding protein (TRBP), a protein that is part of the RNA-induced silencing complex (RISC) involved in the RNA interference (RNAi) pathway [36]. TRBP comprises three dsRNA binding domains (dsRBDs). The positioning of two of them onto short interfering RNA (siRNA) has been investigated by a combination of NMR, electron paramagnetic resonance (EPR) and smFRET. Intermolecular nuclear Overhauser effects (NOEs) between both individual dsRBDs and the siRNA revealed a multiregister binding on the 19 base pair siRNA. Together, NOEs, RDCs and EPR made it possible to identify two species in the dsRBD12 double domain construct, which were both populated. Single molecule FRET was then used to quantify the relative populations of species 1 and 2 using one fluorescent label on the siRNA, and one on the protein. Two FRET populations were clearly visible in the histograms and characterized using recurrence analysis, making it possible to specifically select one population rather than the other [37]. The obtained FRET efficiencies were in agreement with the positions obtained from the combined NMR/EPR analysis, and led to the conclusion of an approximately 1:1 stoichiometry between the two species, independent of whether symmetric or asymmetric siRNA was used [38].

A two domain system comprising extensive dynamics also within the bound complex is the two domain construct 627-NLS of the influenza polymerase protein PB2. The influenza polymerase is composed of three proteins: PA (polymerase acidic protein), PB1 and PB2 (polymerase basic proteins). In the course of the replication cycle, the viral polymerase has to be transported into the nucleus of the infected cell, where it steals short 5′ capped RNA from the host [39] to equip its own mRNA and prepare it for translation back in the cytoplasm. While PA and PB1 are imported into the nucleus as a complex, PB2 is imported separately with the help of a nuclear localization sequence (NLS), which is located in its C-terminal NLS domain. Although the NLS domain has been crystallized with the import receptor Importinα [40] and the two-domain subunit 627-NLS has been crystallized in a compact conformation [41], the actual binding mechanism of 627-NLS to Importinα remained unclear until solution state experiments were performed: Already ^1^H-^15^N correlation spectra revealed twice as many peaks as there were amino acids within the 627-NLS construct, suggesting two interconverting states in slow exchange. One set of the peaks superimposed with the spectra of the 627 and NLS domains individually and was interpreted as an ‘open’ conformation in contrast to the compact conformation observed in the crystallographic structure. Indeed, chemical exchange saturation transfer (CEST) experiments revealed the interconversion between those two states on a time scale of 50 s^−1^ at a temperature of 25 °C, where the open and closed conformations are populated approximately at a ratio of 1:1. This was confirmed by single molecule FRET experiments, where one label was placed on the NLS, and the other one on the 627 domain. Two separate FRET populations were observed and interconverted on a time scale slower than the diffusion time through the observation volume, in agreement with the time scale identified by CEST. The open population deviated from the so-called ‘static FRET line’ describing a linear dependence between intensity based FRET efficiency and donor fluorescence lifetime, and suggesting a rapid sampling of different positions by the 627 and NLS domains, in qualitative agreement with the signature obtained from the NMR chemical shifts and the resulting multiconformational model describing the open state of 627-NLS. Notably, while the Importinα: 627-NLS complex was too large to be observed by NMR, the smFRET experiments made it possible to measure the interaction, which shifted the equilibrium of 627-NLS toward the open conformation, an observation that was also confirmed by small angle X-ray scattering (SAXS) experiments [42]. While the open conformation is thus essential to binding to Importinα and transport into the nucleus, the closed conformation is indispensable for viral polymerase function [43,44], although polymerase conformations have been identified in which 627 and NLS domains are dislocated from each other [45].

**Figure 2:**
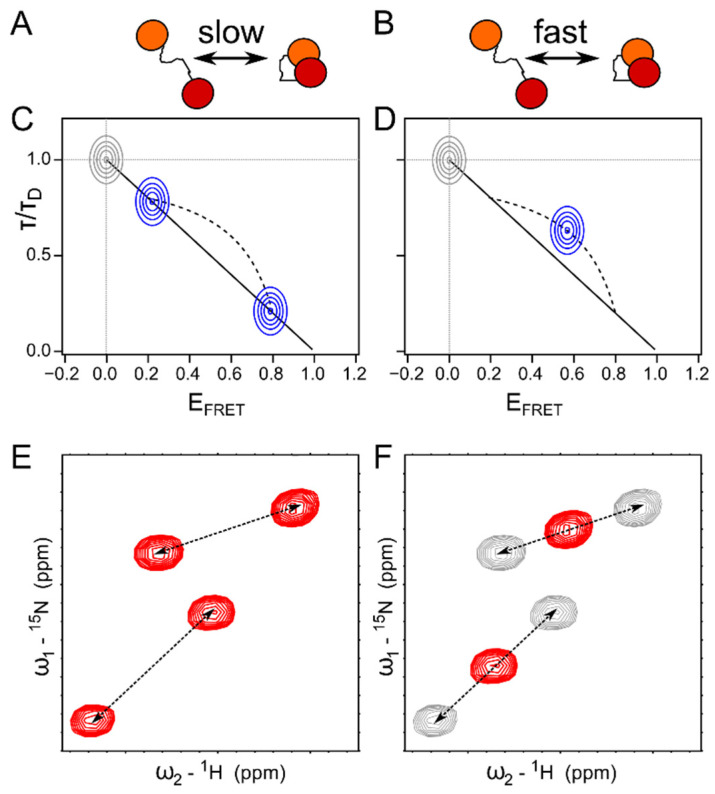
Two-domain interaction in slow (>milliseconds) and fast (submicrosecond) exchange. Schematic of the two limiting cases of slow (**A**) and fast (**B**) exchange for a two-domain protein. (**C**,**D**) Signature of single molecule fluorescence lifetime (τ) versus FRET efficiency (E_FRET_) histogram of a protein of two different states in case of slow (**C**) and fast (**D**) exchange. Shown are donor lifetimes (τ) normalized with respect to the donor only lifetime (τ_D_) in the absence of an acceptor fluorophore. The grey peak reflects the molecules that are not labelled with an acceptor. The blue peaks reflect the peaks corresponding to the double labelled two-domain protein in the different exchange regimes. Note that all peaks are visible simultaneously in a FRET experiment. Solid line: τ versus E_FRET_ dependence for a static molecule. Dashed line: τ versus E_FRET_ for a molecule in two different states (τ/τ_D_ = 0.2 or 0.8, calculated as described in ref. [49]). The position of the blue population in (**D**) depends on the percentage of open and closed conformations in the equilibrium. (**E**,**F**) Signature of a ^1^H-^15^N HSQC spectrum of a protein with two different states in case of slow (**E**) and fast (**F**) exchange. Dashed arrows connect NMR peaks that belong to the same N-H bond in the protein in slow exchange (**E**). (**F**) The red peak illustrates the position of an observed peak for a protein in fast exchange between the two conformations (limiting cases in grey). The exact position of the peak between the two limiting states depends on the percentage of each state populated. Grey peaks are not visible in an experimental spectrum and are shown for illustrative purposes.

A different multidomain system, the U2 auxiliary factor (U2AF), involved in the human spliceosome, is comprised of a large (U2AF65) and small subunit (U2AF35), both of which recognize pre-mRNA through several RNA recognition motif (RRM) domains. U2AF65 comprises three RRM domains, one of which, the U2AF homology motif (UHM), is atypical. Combined NMR (PREs, RDCs) and SAXS studies of the two first RRM domains (RRM1 and 2) revealed that the linker between these two domains samples a conformational ensemble, leading to a continuum of conformations connecting a ‘closed’ and an ‘open/detached’ state [46]. smFRET experiments with one label placed on each RRM domain confirmed the fast exchange between the open and closed states. Rather than showing two separate FRET peaks in the smFRET histogram, a single peak at a FRET efficiency (E_FRET_) >0 was observed; its relationship with respect to the donor fluorescence lifetime indicated a fast averaging regime, deviating from the above-described ‘static FRET line’. Bi-exponential fitting of the experimental fluorescence lifetimes (τ) made it possible to extract the parameters (lifetime and E_FRET_) of the limiting states, which were found to be in agreement with previously determined open and closed states. This allowed the authors to draw a ‘dynamic FRET line’ on which the measured population is shown in the E_FRET_ versus τ histogram (Figure 2C). This analysis also allowed determination of the fraction of time that the protein spends in the open and closed states, and demonstrated that the binding of U2AF35 to the UHM ligand motif (ULM) in U2AF65 led to a shift in the conformational ensemble toward the open state [47]. This open state was also determined to bind the pre-mRNA [47], and thus explains why U2AF35 enhances the affinity [48]. The binding interface between U2AF65 and U2AF35 was, in turn, again determined by NMR spectroscopy. ^15^N spin relaxation showed that ULM and RRM1 of U2AF65 tumbled together with the U2AF35 UHM when free in solution, while RRM2 of U2AF65 tumbled independently—a behavior that changed in the presence of pre-mRNA, where all domains exhibited a common rotational correlation time. PREs eventually allowed determining the relative orientation of the domains with respect to each other in the unbound and RNA-bound state [47].

These three examples showcase different exchange regimes of multidomain proteins and their binding with their respective signatures in NMR as well as smFRET (Figure 2). When in slow exchange (>milliseconds), separate NMR as well as FRET peaks are visible for two states, e.g., open and closed, sampled by a two-domain protein. The relationship between the FRET efficiency and the fluorescence lifetime corresponds to a static distance between the donor and acceptor fluorophores, provided the two states are static in themselves (Figure 2A,C,E). When in fast exchange (submicrosecond), one NMR peak is observable with a chemical shift reflecting the percentage of open/closed conformation compared to the limiting states. smFRET shows one FRET population under these circumstances as well, albeit with a FRET versus fluorescence lifetime dependence reminiscent of a dynamic averaging between the two states (Figure 2B,D,F) [49]. These two-domain protein examples thus demonstrate how the two techniques can complement each other on different time scales.

## 3. Large Intrinsically Disordered Proteins

### 3.1. Partially Folded Proteins

Although multidomain proteins connected by intrinsically disordered linkers can also count as ‘partially folded proteins’, many proteins actually comprise extensive intrinsically disordered domains that may themselves contain transient secondary structures or fold upon binding. One example that was studied using both NMR and smFRET relatively early on is the adenovirus early region (E1A) oncoprotein. E1A interferes with cellular processes by hijacking the cellular machinery for transcription regulation through interaction with the CREB binding protein (CBP, CREB: cyclic-AMP response element binding)/p300 and the retinoblastoma protein (pRb). However, E1A is a nearly entirely disordered protein that comprises transiently folded elements and can partially fold upon binding to its host interaction partners [50,51]. Short linear motifs embedded in the different domains seem to mediate the various interactions of E1A [52]. Both its N-terminal as well as its first conserved region (CR1) have been shown to bind to the TAZ2 domain of CBP/p300 by NMR titrations [50]. While intermediate exchange of the interaction between the E1A N-terminus and TAZ2 prevented structural analysis by NMR, CR1 binding to TAZ2 was in slow exchange and demonstrated coupled folding and binding of the otherwise unfolded CR1, making it possible to determine the structure of the CR1:TAZ2 complex, and leading to the conclusion that the N-terminus of E1A binds to a different surface on TAZ2. pRb was shown to bind to both CR1 and CR2, and led to coupled folding and binding of CR2, while the binding site in CR1 was adjacent to the one occupied by pRb and did not induce folding. Indeed, the formation of a ternary CR1:TAZ2:pRb complex could be observed by NMR, although this led to severe line broadening [50]. In a cellular context, the N-terminus, CR1 and CR2 are not isolated from each other, and tertiary complex formation of E1A, CBP/p300 (TAZ2) and pRb can only really be understood if considered in presence of all relevant subdomains of E1A. Given that this longer construct of E1A is very aggregation prone, and ternary complexes lead to NMR line broadening, Ferreon et al. studied the ternary interactions by single molecule fluorescence [53]. Fluorescence anisotropy revealed very high affinities (K_D_ < 25 nM) for the individual interactions, making this protein system ideal for single molecule FRET approaches, where extremely low concentrations of the labelled protein (in the picomolar range) need to be used [10,32]. Constructs comprising the N-terminus, CR1, CR2, as well as combinations of two or all three domains, were generated and fluorescently labelled at positions surrounding the known interaction sites. Interestingly, all interactions showed signatures of slow exchange compared to the diffusion time of the labelled molecules inside the confocal detection volume (usually on the order of milliseconds) [54], leading to two separate FRET peaks for the bound and the unbound states. Titrations with one or two interaction partners of the different E1A constructs were then used to extract affinity constants, revealing positive as well as negative cooperativity, depending on which domains of E1A were present [53]. Those cooperative interactions were partially explained by the NMR titrations performed previously in the presence of only the CR1 domain [50], and more recent NMR studies suggest the involvement of additional CBP/p300 domains in the interaction with E1A [55,56].

This example shows smFRET can complement molecular NMR studies on IDPs in case of signal broadening due to intermediate exchange on the NMR chemical shift time scale, but also how different concentration regimes used in NMR and smFRET allow the study of aggregation prone proteins. Indeed, even proteins related to aggregation disease can often be studied under single molecule conditions [57,58,59,60], while readily formed aggregates or fibrils are accessible by (solid state) NMR or electron microscopy at atomic resolution [61,62,63,64].

The lack of NMR peaks or assignment can further motivate the investigation of intrinsically disordered protein complexes by single molecule fluorescence, and has shown great benefit in the analysis of the activation mechanism of cyclin-dependent kinases (Cdks). The disordered protein p27 is involved in phosphorylation-dependent Cdk signalling pathways and engages in a complex with Cdk2 and cyclin A in a partially folded conformation [65,66], thereby inhibiting the kinase [67]. A combination of biochemical, cell-biological, and NMR studies indicated that a priming phosphorylation event of p27 on Y88 by a different kinase weakened its binding to Cdk2 through a partial release of a 3_10_-helix, allowing phosphorylation of p27 T187 to occur by Cdk2 [68] and giving rise to the subsequent degradation of p27, rendering the kinase fully active [67]. Indeed, another phosphorylation event at position Y74 seems to be involved in this priming process [69] and has shown to completely release p27 from binding to Cdk2 as demonstrated by NMR chemical shift titrations [70]. As Y88 and Y74 are located in a region that is bound by Cdk2 and only releases upon phosphorylation of the two residues, this raises the question of their accessibility to kinases. NMR backbone assignments being available only for part of p27, this question was addressed by single molecule fluorescence approaches. Fluorescence anisotropy recorded for several labelling sites of p27 on the single molecule level revealed the presence of two states, one with faster (lower anisotropy) and one with slower (higher anisotropy) dynamics, suggesting that the kinase interacting domain (KID) of p27 is not continuously bound to the Cdk2/cyclin A complex. The ratio between these two states was shown to be modulated as a function of Y88 and Y74 phosphorylation, depending on the position of the fluorescent label sensing the dynamic motion of the protein chain, in agreement with the release of both phosphorylated sites from Cdk2 as observed by NMR. smFRET of p27 labelled with a donor and an acceptor fluorophore for smFRET revealed a very broad population again in agreement with the presence of a minor state in slow exchange with the major state, as determined by photon distribution analysis (PDA) [71]. Interestingly, both states show inherent fast dynamics as inferred from their deviation from the static FRET line and fluorescence correlation spectroscopy. The observed minor state was interpreted as dislocated from Cdk2, and increased significantly upon phosphorylation of Y88, in agreement with results obtained from fluorescence anisotropy and NMR chemical shift titrations. This led to the creation of a model by which transient liberation of p27 allowed phosphorylation of Y88 and Y74 and subsequent activation of the kinase [70]. Recent approaches using single molecule FRET have elucidated the long-range conformational changes of p27 upon interaction with Cdk2/cyclin A and have revealed a compact conformation in the unbound state that extends when bound [72], nicely complementing the NMR-observed coupled folding and binding [66].

### 3.2. Disordered Protein Complexes

Although binding induced folding or conformational selection are frequently observed for IDPs and lead to a folded or partially folded state in the complex, a number of IDPs do not seem to fold when engaged in an interaction with their binding partners. The study of those intricate protein systems has also benefited from the combined use of single molecule fluorescence and NMR.

**Figure 3:**
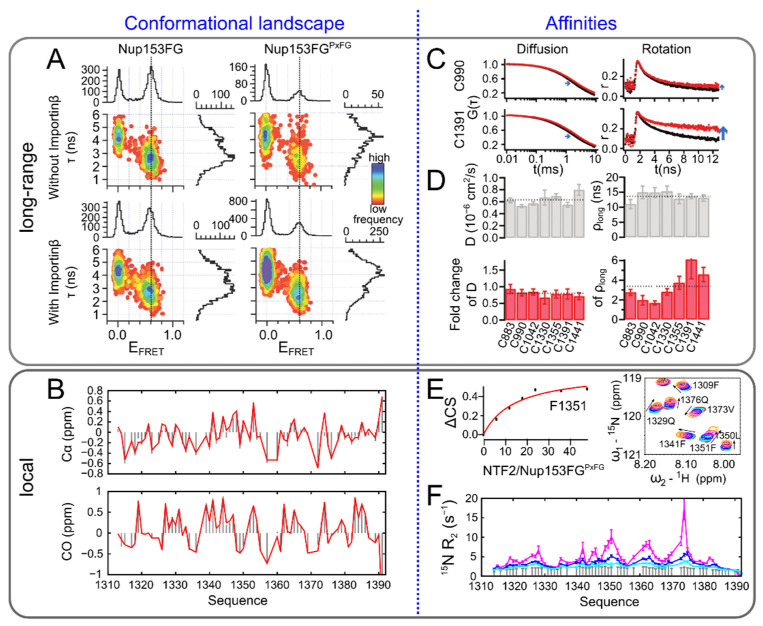
Complementarity of single molecule fluorescence and NMR on the example of the nucleoporin Nup153. (**A**) The FG-domain of Nup153 (Nup153FG) and its shorter variant (Nup153FG^PxFG^) analyzed by single molecule fluorescence. Shown are fluorescence lifetime (τ) versus FRET efficiency (E_FRET_) histograms in the absence (**top**) and presence (**bottom**) of the NTR Importinβ showing no difference in long-range conformational dynamics upon binding [88]. (**B**) Cα and CO secondary chemical shifts of Nup153FG^PxFG^ in the absence (grey bars) and the presence (red lines) of the NTR NTF2 (nuclear transport factor 2) showing no difference in the local conformational propensities upon binding. (**C**) Diffusion (FCS, G(τ)) and rotation (time resolved anisotropy, r) experiments of Nup153FG in the absence (black) and presence (red) of Importinβ from samples labelled at two different positions (see left, Cys 990 and Cys 1391). (**D**) Quantification of the diffusion time (D) and the segmental rotational correlation time sensed by the fluorescent dye (ρ_long_) of Nup153FG alone and in the presence of Importinβ. Shown are values for different labelling sites (bottom axis). Dashed lines correspond to the average between the different labelling sites. [22]. (**E**) Chemical shift difference (ΔCS) of a phenylalanine within Nup153FG^PxFG^ along a titration with NTF2 (ratio of the two proteins is shown on the bottom axis, 80 μM Nup153FG^PxFG^ was used for 6 to 12-fold excess of NTF2, 60 and 40 μM for 36 and 48-fold excess) with exemplary zoom into a ^1^H-^15^N HSQC spectrum of the interaction. Arrows indicate the movement of the respective peaks (right). (**F**) Transverse relaxation (R_2_) of Nup153FG^PxFG^ alone (grey bars) and in the presence of increasing concentrations of Importinβ. 250 uM Nup153FG^PxFG^ was used and Importinβ was at 0.17, 0.33 and 0.72 fold the concentration of Nup153FG^PxFG^ [88]. Adapted with permission from refs. [22,88].

The nuclear pore complex (NPC), for example, a megadalton sized multiprotein machinery controlling the transport between nucleoplasm and cytoplasm, harbors in its center a high concentration of IDPs constituting a so-called ‘permeability barrier’: Only molecules that can bind to nuclear transport receptors (NTRs) via a nuclear localization sequence (NLS) can transition between the nucleoplasm and cytoplasm through the NPC permeability barrier [73,74]. This happens indeed in a highly efficient manner, taking only a few milliseconds [75,76], and is mediated by the interaction between phenylalanine-glycine (FG) motifs within the IDPs, called FG-nucleoporins, and NTRs [73,74]. The molecular properties of this interaction have been elucidated by X-ray crystallography [77,78,79], NMR [80], and molecular dynamics simulation [81,82,83] of NTRs with small FG motif peptides. How specific binding between FG motifs and NTRS, of which affinities different by orders of magnitudes have been observed [84,85,86,87], and rapid transport could be reconciled had, however, long remained enigmatic. While single molecule fluorescence approaches (FCS and fluorescence anisotropy) are able to detect efficient binding between FG-nucleoporins and NTRs, differences in smFRET, such as compaction or extension of the disordered chain, could never be observed upon interaction of the nucleoporin Nup153 with NTRs. Moreover, the chain dynamics seemed to be unaffected by the binding events despite a partially significant size (around 90 kDa) of the NTRs involved in the interaction (Figure 3A). These puzzling results could be explained by NMR experiments, measuring the interaction between Nup153 and the NTR Importinβ at amino-acid resolution. Chemical shift titrations based on ^1^H-^15^N HSQC spectra revealed a very local interaction between the FG-nucleoporin and Importinβ, involving only the FG motifs and their immediate surrounding or even an individual phenylalanine not followed by a glycine, while the overall conformational sampling of the FG-nucleoporin remained unaffected as inferred from a comparison of the ^13^C chemical shifts in the bound and the unbound state (Figure 3B). Rapid exchange (faster than around 10 μs) between the bound and the unbound state of the FG motifs characterized the interaction and identified a residue-specific affinity constant of only a few millimolar (Figure 3E,F), suggesting that binding to and unbinding from an individual motif could be very efficient [88]. This observation has indeed been confirmed by an independent NMR study [89]. Although the binding of the NTR to the entire intrinsically disordered region showed much higher affinities, and stopped flow fluorescence as well as molecular dynamics simulations suggested very rapid association, millimolar local affinities suggest that speed may be ensured from rapid binding and unbinding, and specificity from the multiplicity of the binding motifs [88].

Many FG-nucleoporins seemed to bind to diverse nuclear import and export receptors using the same mechanism, supporting the case of a general binding approach [88]. It has, however, become clear that another binding mechanism might exist, characterized by changing FRET efficiencies between the unbound and NTR-bound FG-nucleoporin Nup214 [90] and by a potentially much more extended interaction site on the FG-nucleoporin as discovered by X-ray crystallography [91]. Although solution state atomic data provided by NMR are not available for this complex, these data support the proposed presence of distinct functions of the cytoplasmic Nup214, as compared with other FG-nucleoporins that reside in the central channel of the permeability barrier.

An interesting observation from NMR and fluorescence approaches investigating FG-nucleoporins has been an apparent preference of different NTRs for specific FG motifs (NMR, Figure 3E,F, [88]) or regions within the FG-nucleoporin (fluorescence anisotropy, Figure 3C,D [22]): ^15^N Transverse relaxation of the FG-nucleoporin Nup153 in the presence of the NTR Importinβ depended on the position of the FG repeat, suggesting a different percentage of bound Importinβ at the different positions [88]. Fluorescently labelled Nup153 showed slowed diffusion times in the presence of Importinβ independently from the labelling position, while segmental rotational tumbling, investigated through time resolved fluorescence anisotropy, depended on the labelling site, making a case for specific affinities along the nucleoporin sequence [22]. Different specific ^15^N transverse relaxation rates and time resolved fluorescence anisotropies were found in the presence of the NTRs transportin 1 (TRN1) and nuclear transport factor 2 (NTF2) [22,88]. Although quantitative comparison of these observations is challenging, both types of experiments point toward an impact of the context the FG motifs are embedded in on binding [92]. FG-nucleoporins have, in the meantime, been shown to be able to associate into liquid-like condensates, which can enrich NTRs [93], a mechanism related to the previously observed hydrogel formation of FG-nucleoporins and their permeability barrier-like character [94,95].

A different intricate protein system engaged in binding of two different IDPs with each other is the linker histone H1.0 and its chaperone prothymosine α (ProTα). H1.0, involved in chromosome condensation by binding to the nucleosome [96], is largely disordered comprising only a small globular domain, and is highly positively charged. Its histone chaperone, ProTα, proposed to aid in the incorporation of H1.0 into the nucleosome [97], is of opposite charge and entirely disordered. smFRET experiments have in the past demonstrated the sensitivity of ProTα long-range interactions to surrounding charges by varying the salt concentrations contained in the buffer [98]. A compaction of donor/acceptor doubly labelled ProTα was also observed when unlabeled H1.0 was present in the experiment, supporting the case for a charge screening similar to what had been observed in high ionic strength buffers [99]. The transition towards a more compact state of ProTα was, however, not gradual; rather, the compact conformation existed as a separate FRET peak, increasing in population relative to the FRET population of the unbound ProTα and therefore suggesting a specific interaction with histone H1.0 in slow exchange with respect to the diffusion time of the protein complex through the observation volume (milliseconds) of a confocal setup [54]. Intermolecular FRET between ProTα and H1.0 confirmed a 1:1 interaction stiochiometry at which both proteins would remain disordered and the titration data between the two molecules could be used to calculate affinity constants, which were in the picomolar range, but dependent on buffer ionic strength [99]. While stopped flow association kinetics suggested exchange rates that would lead to the observation of two separate bound and unbound peaks of ProTα in an NMR spectrum [100], the signature of the interaction by NMR pointed towards a dynamic complex, as also suggested by smFRET, fluorescence lifetimes and nanosecond fluorescence correlation spectroscopy (nsFCS), but in fast to intermediate exchange on the chemical shift time scale [99]. Indeed, the observed NMR signature seemed in much better agreement with isothermal titration calorimetry (ITC) data measured on the same complex and also proposing a 1:1 stoichiometry. As a result, fluorescence labelling was first held responsible for the apparent discrepancy between smFRET and NMR concerning the observed affinities and exchange rates [97]. Apart from different labelling requirements for smFRET and NMR experiments, one major experimental difference between the two techniques is the concentration regimes used. An artificial increase of the protein concentration in the single molecule FRET experiment by addition of excess unlabelled protein elegantly demonstrated that the interaction could be shifted towards intermediate to fast exchange also in the smFRET experiment. This unusual behavior was explained by the formation of a transient ternary complex involving two times H1.0 and supported by extensive fluorescence stopped flow kinetics, smFRET recurrence analysis [37], and smFRET kinetics using immobilized ProTα, and yielding rate constants in agreement with the observed NMR signature [100].

It is of note that both NMR and smFRET succeeded in observing a protein complex between two intrinsically disordered proteins and smFRET showed that the globular domain of H1.0 only contributed weakly to the interaction at single molecule (picomolar) concentrations of ProTα. Indeed, although titration of the globular domain of H1.0 into ^15^N labelled ProTα resulted in the same interaction signature along the amino acid sequence of ProTα as compared to addition of full length H1.0, the observed chemical shift perturbations were less pronounced, supporting a similar conclusion at higher protein concentrations [99].

In fact, although potential concentration effects need to be analyzed carefully, the different concentration regimes of single molecule FRET and NMR can be an advantage when analyzing protein:protein interactions. Together smFRET and NMR offer a vast dynamic range for the analysis of different affinity ranges, allowing to access both strong and weak binding events. Thanks to the resolution of NMR, extremely weak binding can also be discovered in the presence of additional strong interactions [101].

## 4. Liquid–Liquid Phase Separation

An extreme case of dynamic interaction networks exhibited by IDPs lies in liquid–liquid phase separation, which is now understood as an important cellular mechanism for nonmembranous compartmentalization. Fluorescence imaging, together with fluorescence recovery after photobleaching (FRAP), have probably been the two main techniques to be used to investigate phase separated systems. While FRAP provides molecular insights into diffusion times when analyzed quantitatively and under the assumption that the boundary conditions of the fit are properly defined [102], performing both single molecule fluorescence and NMR in the protein dense phase is challenging.

Although phase separating proteins are at high concentrations inside the condensed phase, their rotational tumbling time can be very slow due to the high viscosity of their surroundings and the interactions undertaken within the condensed phase, leading to severe NMR line broadening [103,104,105]. NMR has nevertheless yielded important insight into condensed phases specifically enriched in the NMR tube by centrifugation [105,106,107] and novel developments to tackle this particular protein environment are continuously emerging [108,109,110] and reviewed elsewhere [111].

### 4.1. Ensemble Fluorescence Combined with NMR

Aside from classical fluorescence imaging and FRAP, fluorescence spectroscopy approaches used in the study of phase separated systems so-far concerned mainly the measurement of fluorescence anisotropy decays [103,112] or fluorescence correlation spectroscopy based approaches [113,114]. Although time resolved fluorescence anisotropy on tau protein has shown a decrease in rotational correlation time within the liquid dense phase interpreted as a de-compaction of the protein within the condensed compartment [112], the opposite was the case for liquid droplets formed by the measles virus replication machinery: In the phase separating system, where both liquid and condensed phases coexisted, NMR signal could only be obtained from the liquid phase, testified by transverse relaxation rates reporting on rotational motion and NMR diffusion experiments that were unchanged between a dilute protein sample and at phase separating conditions. In contrast, fluorescence signal was obtained from both liquid and condensed phases and time resolved anisotropy significantly increased under phase separating conditions, where measles phosphoprotein and nucleoprotein were mixed at physiological salt concentrations. Experiments at high salt concentrations preventing phase separation, revealed the impact of molecular interaction on time resolved anisotropy and thus led to the conclusion that rotational motion of both phosphoprotein and nucleoprotein was slowed down inside the condensed phase—an observation that is in agreement with rotational correlation times leading to increased line broadening in an NMR experiment. However, observation of the NMR signal originating from the liquid phase in a coacervate made it possible to indirectly observe the condensed phase, and led to the conclusion that the stoichiometry between nucleoprotein and phosphoprotein within the condensed phase is not fixed, but can vary according to the availability of both proteins. This observation is in agreement with a tetrameric configuration of the phosphoprotein, possessing three main interaction sites with the nucleoprotein on each monomer [103].

One way to measure protein concentrations specifically within condensed and liquid phases, and therefore, to directly determine the ratios between the different components of the condensed phase, is to use fluorescence correlation spectroscopy (FCS). FCS has thus contributed to the construction of so-called ‘binodals’, determining the coexistence of liquid and condensed phases [113,114]. This approach, in conjunction with the extraction of diffusion times from FCS experiments, demonstrating a dramatically reduced mobility within the condensed phase, has been used in conjunction with NMR on the low complexity region (LCR) of the heterogeneous nuclear ribonucleoprotein (hnRNPA1). ^15^N transverse relaxation of the hnRNPA1 LCR revealed locally increased rates, coinciding with the presence of hydrophobic amino acids and revealing interactions that were also observed as nuclear Overhauser effects, despite the intrinsic disordered nature of the protein [113]. These findings were interpreted in the context of the recently postulated stickers and spacers model [115], supported by specific mutations within the protein sequence, according to their molecular dimensions, as measured by SAXS, and their phase separating behavior [113].

### 4.2. Single Molecule FRET Combined with NMR

While FCS is often counted among single molecule techniques as it is performed on very small protein concentrations, studies employing single molecule FRET on liquid–liquid phase separation remain sparse [116,117,118,119]. Successful initiatives to combine smFRET and NMR have, however, been undertaken on the example of nucleophosmin (NPM1), a chaperone of the nucleolus known to bind proteins enriched in arginines (so-called R-motifs). A whole cell pull-down experiment that first identified binding partners and ensemble fluorescence anisotropy was used to measure binding of R-motif containing peptides to NPM1. Interestingly, titration of a fluorescently labelled NPM1 construct with an R-motif peptide showed two anisotropy transitions, the second of which coincided with liquid–liquid phase separation of the system visualized by fluorescence microscopy. In an NMR chemical shift titration, affinities of different strengths were identified all along the sequence of NPM1_1–130_ comprising the N-terminal heptamerization domain as well as two acidic tracts, the second being of higher flexibility that is compromised under phase separating conditions, demonstrated by ^15^N longitudinal relaxation and transverse dipole-dipole/CSA cross-relaxation. smFRET of the doubly labelled NPM1_1–130_ was performed to investigate conformational changes upon phase separation [117]. In a followup study, the authors demonstrated that in addition to phase separation in the presence of R-motif proteins or RNA, NPM1 comprising its central IDR could also self associate into separated phases, mediated by one of its acidic and basic tracts. This salt-dependent self-interaction was identified using NMR chemical shift titrations and diffusion experiments, smFRET of labelled NPM1 as well as SAXS [118]. smFRET experiments have indeed also made it possible to follow up on the observation of an equilibrium between the folded pentameric and disordered monomeric forms of NPM1, first discovered by NMR [119,120]. This equilibrium being salt dependent, time scales of folding and pentamer assembly have been investigated using intra- and intermolecular FRET at high salt concentrations. A comparison of the obtained time scales from smFRET experiments with folding rates determined by circular dichroism (CD) spectroscopy indicated a relatively rapid compaction of NPM1 before the actual folding transition. Disassembly, initiated by a transition from high to low salt buffer, revealed a rapid unfolding at time scales comparable between smFRET and CD experiments. This initial unfolding was followed by an intermediate FRET state, which was interpreted as an oligomeric disordered state, before reaching the final unfolded monomeric state [119]. Mutants mimicking phosphorylation of NPM1 seemed to favor unfolded intermediate states, precluding heptamerization and folding, an effect that could be counterbalanced in the presence of the binding partner and tumor suppressor Arf [119]. These results are in agreement with and explain the kinetic pathways related to previously published NMR on the same phosphorylated and unphosphorylated system [120].

## 5. Towards a Quantitative Combination of smFRET and NMR

The examples presented in the previous chapters make it clear that analyzing intrinsically disordered protein systems with both NMR and single molecule fluorescence spectroscopy can be of real benefit, and that the two techniques complement each other in numerous ways. This realization led to the first attempts to combine smFRET and NMR, not only qualitatively for the investigation of IDPs and their interactions, but also to derive conformational ensembles for IDPs in agreement with the parameters obtained from the two techniques.

**Figure 4:**
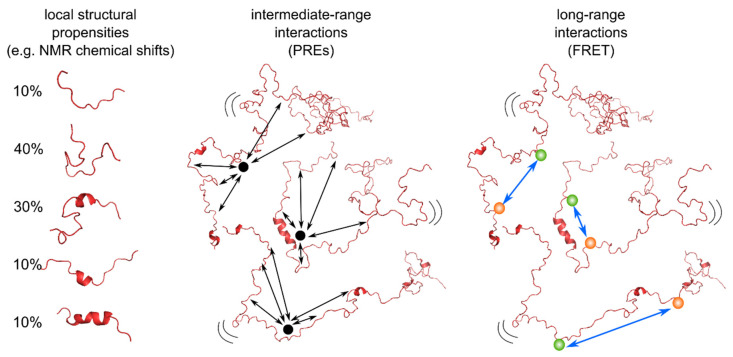
Integration of parameters from NMR and single molecule FRET to derive local structural propensities, intermediate-range interactions and long-range interactions. NMR chemical shifts or RDCs, for example, can inform about population of transient secondary structures. PRE labels (black points) attached to a specific position within the protein (usually a cysteine) can be used to measure the distances between that label and every N-H bond within the protein backbone at distances of up to around 2.5 nm [128]. FRET labelled proteins use an energy donor fluorophore (green) and an acceptor (red), both usually attached to specific cysteines using maleimide chemistry. Distances of up to 10 nm can be measured between the two fluorophores [32].

NMR chemical shifts (CS), residual dipolar couplings (RDCs), scalar couplings as well as paramagnetic relaxation enhancements (PREs) have, in the past, been used to derive conformational ensembles describing IDPs (reviewed in [11]). Those parameters have often been used in conjunction with small angle scattering techniques, providing additional information on the overall extension of the disordered protein ensemble [42,46,88,121,122,123]. Single molecule fluorescence, on the other hand, and in particular smFRET, providing access to specific distances in the protein chain due to site specific attachment of the fluorophores, has mainly obtained distances assuming the behavior of the protein chain according to a polymer chain model [15,18,98,124]. The advantage of this approach is that polymer models provide defined dependencies between end-to-end distances (R_E_) and radius of gyration (R_G_), leading to comparisons between distances measured using smFRET and SAXS. As such, a lot of effort has been devoted to bringing the two techniques into agreement, as well as to explaining apparent discrepancies [21,125,126,127]. Probably due to the atomic resolution of NMR, making the use of analytical polymer models difficult, quantitative comparisons between smFRET and NMR are only slowly emerging with the aim of explaining local structural propensities, intermediate-range as well as long-range interactions within one conformational ensemble (Figure 4).

A relatively early example for the calculation of conformational ensembles of IDPs using NMR, smFRET and SAXS was performed on urea unfolded ubiquitin [34]. Conformational ensembles were calculated using X-PLOR-NIH [129] with restraints from previously published 419 RDCs [130,131], 253 PREs [132], 71 backbone ^3^J_HNHα_-couplings [133], and a SAXS intensity profile [134]. 400 conformational ensembles of 20 conformations each, together suggested the presence of transient secondary structures and long-range contacts also in the unfolded state of ubiquitin. smFRET experiments were performed on 7 double cysteine variants of ubiquitin labelled with Alexa488 and Alexa594 between which energy transfer was measured on the single molecule level and at denaturing conditions comparable to those used for NMR and SAXS experiments. Interdye distances were extracted from FRET efficiencies and fluorescence lifetimes independently, using a Gaussian chain model, and were in very good agreement with each other. In order to compare the obtained Gaussian chain distance distributions with those obtained from the NMR and SAXS restrained conformational ensembles, the contribution attributed to the fluorescent dyes and their linkers was subtracted from the FRET-distances by assuming them to be represented by an equivalent of 9 amino acids [34]. Although the precise number of amino acids in a Gaussian chain model required to represent fluorescent dyes and linkers in an smFRET experiment is not without controversy [21,34,135], the distance distributions derived from smFRET are in very good agreement with those computed from the NMR/SAXS ensemble [34]. Explicit conformational ensembles of a disordered protein complex have also been calculated using coarse-grained modelling, by which one amino-acid was represented by one bead and agreement with experimental FRET efficiencies was achieved with the help of an adjustable energy term. Dye-linker contributions were approximated by the addition of 5 beads into the conformational model and agreement with respect to the NMR-observed interaction sites was obtained [99].

A different approach was taken to study the N-terminal part of the Sic1 protein (amino acids 1–90), an inhibitor of cell cycle progression in yeast. In order to analyze the global extension of Sic1 in its phosphorylated and unphosphorylated state, SAXS and smFRET were performed on the unlabeled and double-cysteine labelled protein, respectively. A single FRET distance of the protein labelled at the N- and C-terminus showed mildly distinct FRET efficiencies for the phosphorylated and the unmodified Sic1. Although several homopolymer models were used for the analysis of the smFRET data and various analysis methods tested for extracting the global protein extension from the SAXS data (Guinier analysis [136], distance distribution function P(r) [137], molecular form factor [138]), the inferred end-to-end distances and radii of gyration between smFRET and SAXS experiments did not come to an agreement. Since also the ensemble selection methods EOM [139] and ENSEMBLE [20,28] did not yield end-to-end distances in agreement with the smFRET results when only SAXS was used for ensemble calculation, previously published NMR chemical shifts (C_α_, C_β_) and PREs were used in conjunction with the measured SAXS curves to define ensembles using the algorithm ENSEMBLE. The measured FRET efficiency was back-calculated from those ensembles by adding accessible volumes describing the fluorophores and their linkers to every conformer in the ensemble and comparing those to the experimentally obtained value, an approach that has frequently been used for folded proteins [32,140,141,142,143,144]. Sampling of the accessible volume was supposed to occur on a time scale significantly longer than the fluorescence lifetime, justified by Monte Carlo and Brownian dynamic simulations to describe the photon emission process and translational diffusion of the dyes, respectively. Those ensembles were in good agreement with the experimental FRET efficiency, while chemical shifts had to be included into the ensemble determination process explicitly [145].

**Figure 5:**
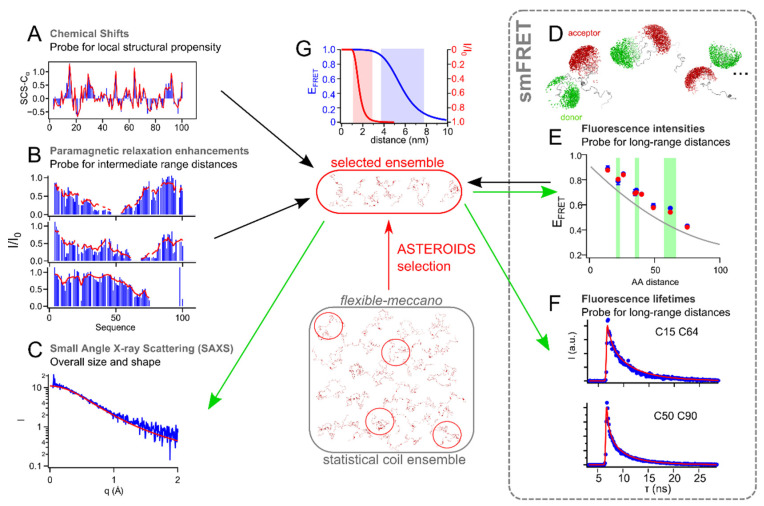
Conformational ensembles in agreement with smFRET (FRET efficiencies, E_FRET_, and lifetimes), NMR (chemical shifts and PREs) and SAXS of P_1–100_. (**A**) C_α_ secondary chemical shifts. Blue: experimental; red: calculated from an ASTEROIDS ensemble selected based on chemical shifts, 6 E_FRET_ and 5 sets of PREs. (**B**) Experimental PREs (blue) and PREs calculated from the selected ensemble (red). Shown are peak intensity ratios. (**C**) Experimental SAXS (blue) and SAXS curve back calculated from the conformational ensemble (red). (**D**) Representation of exemplary conformations of the ensemble and their acceptor and donor accessible volumes. (**E**) E_FRET_ plotted against amino acid (AA) distance between the attached labels. Blue: experimental data and experimental error; Red: E_FRET_ calculated from the selected ensemble; Grey: E_FRET_ expected from random coil. Cross-validated E_FRET_ are above green background. (**F**) Cumulated single molecule lifetime decays (blue) and the decay curves back calculated from the selected ensemble (red). Green arrows point towards data used for cross-validation. (**G**) Dependence of E_FRET_ and PREs (I/I_0_ are shown) on distance. Förster distance = 56 Å, donor lifetime = 4 ns; τ_C_ = 5 *ns* [32,128]. Shaded areas refer to the sensitive regimes of the respective technique. Adapted with permission from ref. [148]. Copyright 2021 American Chemical Society.

A recent study presented an approach to integrate smFRET, NMR and SAXS employing the statistical coil generator flexible meccano [146] in combination with the genetic algorithm ASTEROIDS [26,147] to derive conformational ensembles in agreement with all data sets and of predictive nature [148]. Accessible volume calculations describing the fluorescent dyes site-specifically attached to the protein chain [140,149] were optimized for computation speed, allowing us to calculate fluorophore labelled conformers of large conformational ensembles (10,000 conformers in size), from which subensembles could thus be selected using FRET efficiencies. In silico data (15 FRET efficiencies distributed along the protein chain and 5 sets of PREs) of an IDP comprising a long-range contact (<20 Å) were generated and part of the data used to select ensembles of 200 conformers in size in agreement with all simulated parameters. As a simulated data set was used to benchmark the integration of FRET efficiencies and PREs into multiconformational models, the size of the selected ensemble, the number and distribution of FRET efficiencies along the protein sequence, and the complementary nature of PREs and FRET could be addressed unambiguously: Ensemble sizes on the order of 100 conformers or larger reflected the statistics of the input ensemble from which in silico NMR and FRET data were calculated, and yielded accurate reproduction of the in silico data. If sufficient FRET distances were sampled along the protein sequence, the remaining FRET efficiencies were reproduced with confidence, but failed to predict the in silico PREs and vice versa, demonstrating that only a combination of NMR and smFRET produced reliable ensembles satisfying both long- and short-/intermediate-range information encoded in the conformational ensemble. The authors used experimental NMR data (H_N_, H, C_α_, C_β_, CO chemical shifts and 5 sets of PREs), 9 FRET efficiencies with their corresponding fluorescence lifetimes and one SAXS curve measured on the first 100 amino acids of the measles phosphoprotein (P_1–100_) to test the approach on measured data. All NMR parameters and 6 FRET efficiencies were used to select conformational ensembles using ASTEROIDS. The remaining FRET efficiencies, all fluorescence lifetime decays, calculated from the single molecule FRET data, and the SAXS curve were left apart for cross-validation, which successfully demonstrated the predictive nature of the approach (Figure 5). The time scales of accessible dye volume sampling as compared to the fluorescence lifetimes could also be addressed thanks to the number of FRET efficiencies measured and the experimental data were best reproduced with a sampling longer than the fluorescence lifetime [148]. Overall, this comprehensive study now proposes a versatile toolset for the generation of conformational ensembles describing IDPs based on integrated experimental data that can also be used with complex structural arrangements comprising folded and disordered domains and awaits to be tested with this kind of protein systems.

Combined consideration of NMR (PREs, RDCs, ^15^N spin relaxation) and smFRET (inferred distances) has also been performed in the course of the development of new molecular dynamics (MD) force fields for IDPs, where experimental data have been used to validate the performed simulations on the example of α-synuclein [150]. The same model protein was used to compare protein dynamics obtained from MD simulations to those from experimental NMR (^1^H relaxometry, ^15^N relaxation rates R_1_, R_2_ and ^1^H-^15^N NOEs) and single molecule fluorescence (nanosecond FCS), and revealed protein dynamics at different time scales [35]. With the inclusion of nanosecond FCS, sensitive on a time scale on the order of several tens of nanoseconds to microseconds (and theoretically up to the diffusion time on the order of milliseconds), a regime that is difficult to quantitatively access by NMR, this approach has potential [34,35] and awaits application to other biological systems.

## 6. Conclusions and Perspectives for the Combined Use of Single Molecule Fluorescence and NMR

Single molecule FRET and NMR spectroscopy have been successful individually in analyzing diverse aspects of IDPs for a significant amount of time and it is thus not surprising that many NMR and fluorescence laboratories have teamed up to access previously unexplored features of IDPs by combining the two techniques. Indeed, although mainly qualitative or semi-quantitative, those approaches have already demonstrated at this early stage how different length scales and dynamics can be addressed [38,42,47]. Since single molecule fluorescence and NMR have specific sensitivities, suffer from different limitations and use vastly different protein concentration ranges [53,70,99,100], their combination can be extremely powerful.

While NMR and fluorescence spectroscopy approaches together have already made great impact in the study of liquid–liquid phase separated systems [103,113,117], studying the condensed phase remains challenging for both techniques albeit for different reasons. The development of NMR pulse sequences to access specific properties related to the separation of phases is, however, currently ongoing [108,109,110], and these approaches will no doubt soon be combined with single molecule fluorescence techniques. Poor signal to noise, often related to high background protein concentration, is a common problem in the study of weak interactions by single molecule fluorescence, or when recorded in the cellular environment. Indeed, initial studies showed that smFRET can be measured within the cell [151,152,153], and undertakings to improve signal to noise for those samples could potentially be transferred to liquid–liquid phase separated systems.

The first successful studies combining single molecule FRET and NMR have thus pushed the field to develop methods to study this area of dynamic structural biology quantitatively and devise multiconformational models from both NMR and smFRET that describe the different aspects of an IDP conformational landscape. While various NMR parameters have been used in the past to devise conformational ensembles of IDPs, integrating smFRET into these ensembles is challenging as the contribution of fluorescent dyes and their linkers need to be taken into account. For this, two strategies have been employed: the calculation of end-to-end distances according to polymer models from which the contribution of the dyes and linkers are subtracted in an approximation [34], and the addition of dye accessible volumes into the conformations in an ensemble [145,148], a strategy borrowed from quantitative descriptions of folded proteins by smFRET [32,140,141]. Both approaches have successfully been used to validate ensembles calculated from NMR and SAXS. Given that the sensitivity of smFRET and NMR parameters to different length scales (even considering PREs, see Figure 5) may sometimes prevent cross-validation between the different techniques, a recent study proposed an approach to derive predictive ensembles calculated based on integrated smFRET, NMR and SAXS data [148], which is applicable to complex biological systems.

IDP conformational landscapes are, however, not solely described by their conformational properties, but also by the dynamics underlying the interchange between the different conformers and related to partner binding. Further studies will certainly follow up on the initial strategies presented herein, also applying single molecule fluorescence and NMR to describe the dynamic properties of IDPs [35].

## Data Availability

Not applicable.
